# The challenge of advanced therapies in the contemporary era: first in Europe ECPELLA long-distance transfer—a case report

**DOI:** 10.1093/ehjcr/ytae151

**Published:** 2024-03-21

**Authors:** Francesca Fiorelli, Vasileios Panoulas, Fernando Riesgo Gil, Carl Era, Alexander Rosenberg

**Affiliations:** Department of Cardiology, Royal Brompton and Harefield Hospitals, Guy’s and St Thomas’ NHS Foundation Trust, Harefield Hospital, Hill End Road, Harefield, Uxbridge, UB9 6JH, UK; Department of Cardiology, Royal Brompton and Harefield Hospitals, Guy’s and St Thomas’ NHS Foundation Trust, Harefield Hospital, Hill End Road, Harefield, Uxbridge, UB9 6JH, UK; Cardiovascular Sciences, National Heart and Lung Institute, Imperial College, Dovehouse Street, London SW3 6LY, UK; Department of Cardiology, Royal Brompton and Harefield Hospitals, Guy’s and St Thomas’ NHS Foundation Trust, Harefield Hospital, Hill End Road, Harefield, Uxbridge, UB9 6JH, UK; Capital Air Ambulance, Bristol, BS48 3DP, UK; Department of Cardiology, Royal Brompton and Harefield Hospitals, Guy’s and St Thomas’ NHS Foundation Trust, Harefield Hospital, Hill End Road, Harefield, Uxbridge, UB9 6JH, UK

**Keywords:** ECMO, Impella, ECPELLA, Cardiogenic shock, Long-distance transport, Case report

## Abstract

**Background:**

The use of mechanical circulatory support (MCS) has markedly increased over the last decade, so have the inter-hospital transfers, with the aim of being able to offer advanced heart failure (AHF) therapies and centralizing patients to tertiary centres.

**Case summary:**

In this article, we present the first in Europe long-distance air transfer of a patient supported by veno-arterial extracorporeal membrane oxygenator and Impella (ECPELLA), as a bridge to successful heart transplant. In our case report, a foreign young patient with AHF due to familiar cardiomyopathy required multiple MCS devices to achieve cardiovascular stability. After appropriate planning and multidisciplinary discussion, the patient was transferred on MCS to his country of origin via a fixed-wing airplane, in order to be assessed for heart transplantation. During take-off, the Impella flows temporarily dropped and a suction alarm was displayed; however, this rectified without intervention, and the rest of the flight was uneventful. One month after transfer, the patient underwent successful heart transplantation and remained clinically stable during the 12-month follow-up.

**Discussion:**

Our experience links together the current challenges in the evolving AHF strategies and the increased need for inter-facility cooperation. Both these clinical and logistic challenges appear to lead to possible improved outcomes, after appropriate assessment, training, and accurate planning. Our experience provides useful information on feasibility of long-distance transport of patients supported by ECPELLA in Europe.

**Learning points**

- After extracorporeal membrane oxygenator (ECMO) implant, the patient can develop flash pulmonary oedema due to increased left ventricular filling pressures in the context of increased afterload.
- Appropriate assessment, training, and accurate planning is of paramount importance to safely perform long-distance transport of patients on mechanical circulatory support.

## Introduction

In the last decade, the use of mechanical circulatory support (MCS) in the context of cardiogenic shock (CS) has markedly increased, with the development of different devices to enhance MCS physiology and improve clinical outcomes.^1^

In addition to the increased complexity of MCS strategies, there has been a rise in inter-hospital transfers, with the aim of centralizing patients to tertiary centres and being able to offer advanced heart failure (AHF) therapies such as transplant or durable left ventricular (LV) assist device.^2,3^

We describe our experience with the first in Europe 900 mile air transfer of a patient supported by veno-arterial extracorporeal membrane oxygenator (VA-ECMO) and Impella (ABIOMED Inc., Danvers, MA) (ECPELLA), as bridge to successful heart transplant.

## Summary figure

**Figure:**
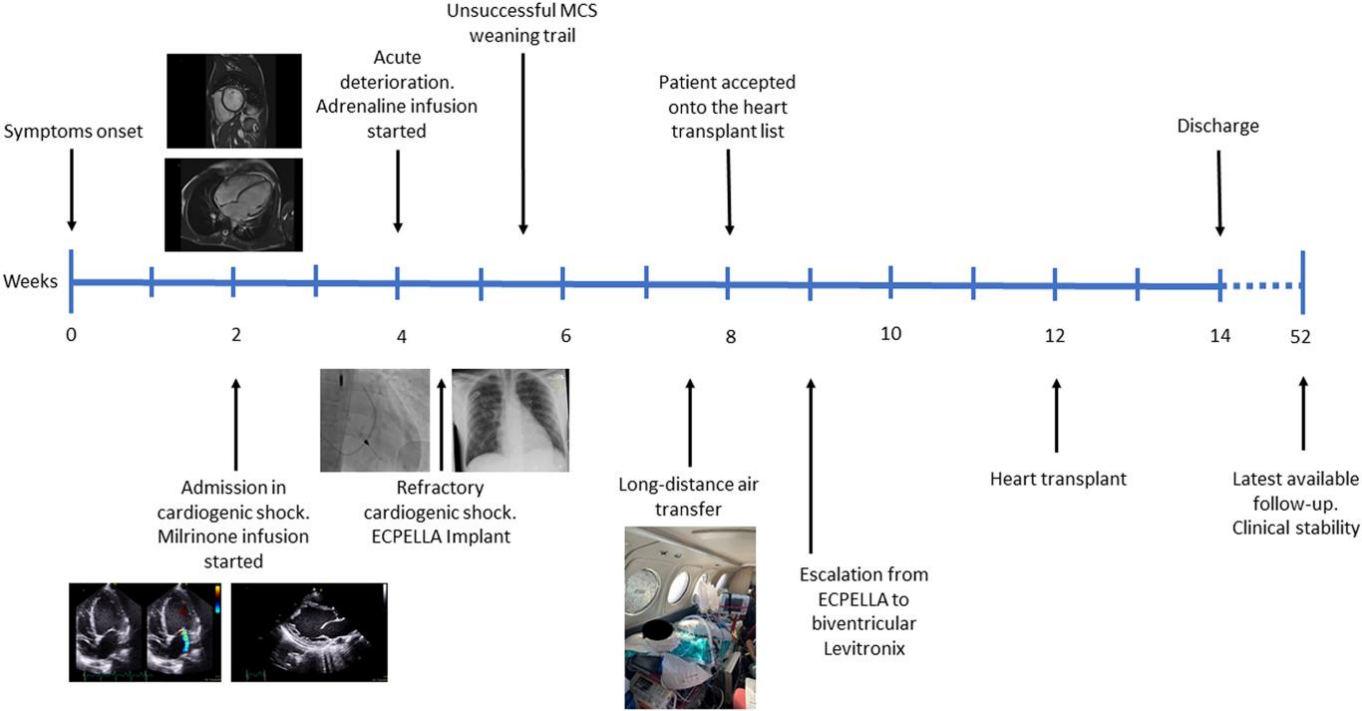


## Case presentation

**Figure 1:**
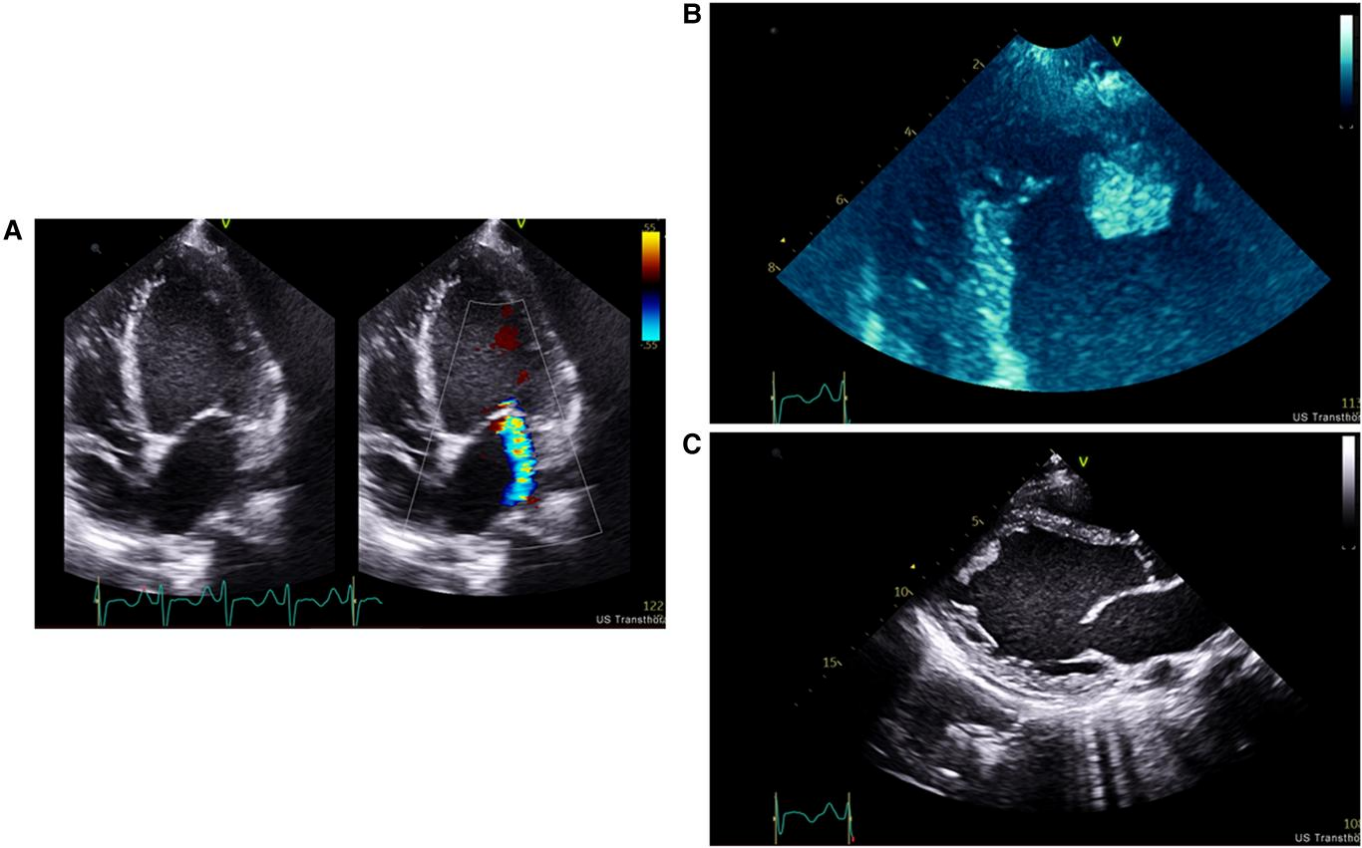
Transthoracic echocardiogram on admission. (*A*) Apical four-chamber view with colour flow Doppler at the level of the mitral valve. (*B*) Apical four-chamber view focused on the left ventricle apex with intra-cardiac thrombus. (*C*) Parasternal long-axis view.

**Figure 2:**
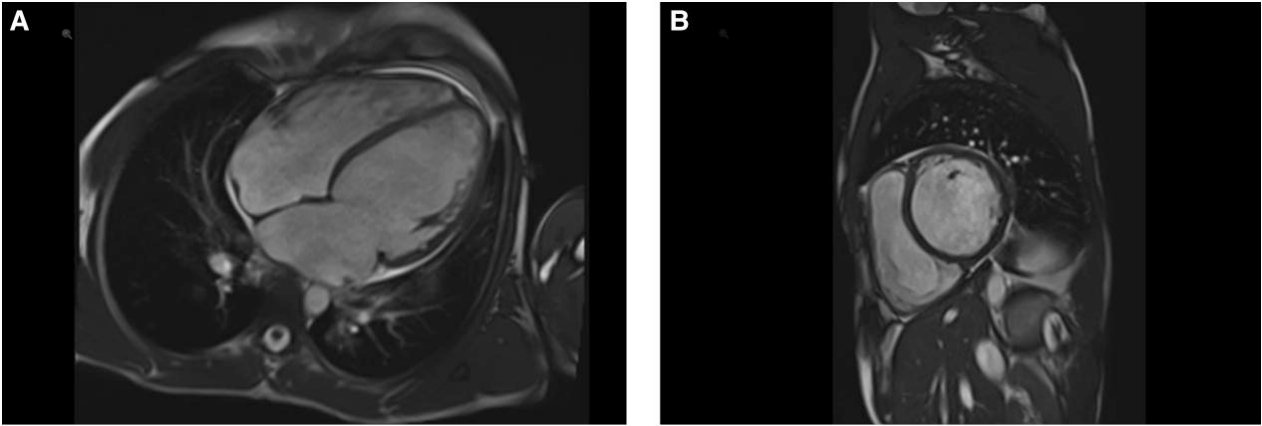
Cardiac magnetic resonance imaging (MRI). (*A*) Four-chamber view. (*B*) Short-axis view.

**Figure 3:**
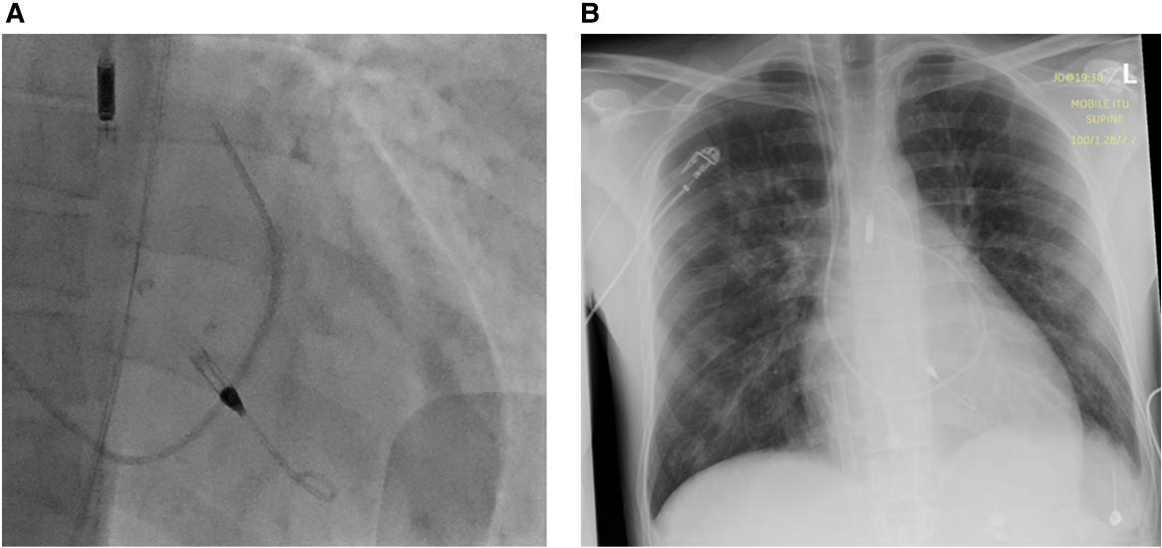
(*A*) Fluoroscopy antero-posterior view. Impella in correct position into the left ventricle (LV). (*B*) Postero-anterior chest X-ray. A 25 Fr multistage VA-ECMO venous cannula from the inferior vena cava with the tip at the level of the right atrium. Impella in correct position into the LV.

A 27-year-old male originally from Poland was admitted to our institution in CS after 2 weeks of worsening dyspnoea. Past medical history was negative for comorbidities; however, it revealed a strong family history of sudden death and cardiomyopathy. Genetic testing confirmed a pathogenic titin mutation. On admission, the patient was euvolaemic with no significant peripheral oedema, no raised jugular venous pressure, or chest congestion on auscultation. He was hypotensive with systolic blood pressure (BP) ∼90 mmHg, mean BP ∼60 mmHg, and with persistent sinus tachycardia at a heart rate of ∼120 b.p.m. Haemodynamically unstable with acute end-organ injury, the patient was started on milrinone infusion at 0.4 mcg/kg/min. The echocardiogram (*Figure 1*) and magnetic resonance imaging (MRI) (*Figure 2*) confirmed severe biventricular dysfunction with LV apical thrombus. The coronary angiogram excluded concomitant coronary artery disease. Invasive assessment with right heart catheterization revealed low cardiac index despite inotropic support, 1.55 L/min/m^2^, with post-capillary pulmonary hypertension (mean pulmonary pressure 33 mmHg, wedge 28 mmHg, right atrial pressure 5 mmHg); however, the end-organ function normalized 1 week after the acute presentation. Despite this initial improvement on a single inotrope, the patient deteriorated into refractory CS after 2 weeks, with progressive hypotension, mean arterial pressure below 60 mmHg, worsening sinus tachycardia, decreased urine output, and rising lactate up to 4.8 mmol/L. Despite additional inotropes and vasopressors and progressive escalation of infusions up to milrinone 0.5 mcg/kg/min, adrenaline 0.1 mcg/kg/min, and noradrenaline at 0.03 mcg/kg/min, the patient kept sliding deeper into CS (stage D CS). Following a rapid multidisciplinary team discussion (MDT), he was escalated to MCS with peripheral VA-ECMO, via right arterial and venous femoral accesses with insertion of 17 and 25 Fr cannulas, respectively. Distal limb perfusion cannula was not inserted due to common femoral artery size ∼10 mm. As soon as the ECMO circuit started, the patient developed flash pulmonary oedema. The bedside echo excluded the presence of aortic regurgitation and showed worsened mitral regurgitation from moderate-severe to now severe and confirmed resolution of the LV apical thrombus; the invasive monitoring showed a wedge pressure of 36 mmHg. Therefore, the patient was promptly treated with Impella CP insertion via the left femoral artery (*Figure 3*). Before Impella insertion, a pigtail catheter was inserted into the LV and confirmed an LV end-diastolic pressure (LVEDP) of 44 mmHg. After Impella insertion, the pulmonary artery (PA) catheter showed a systolic pulmonary pressure of 27 mmHg, diastolic of 17 mmHg, and mean pulmonary pressure at 20 mmHg, the wedge was 16 mmHg, and the right atrial pressure 9 mmHg. Despite the rapid escalation of events, the patient did not require intubation and was maintained alert and self-ventilating throughout the subsequent intensive therapy unit (ITU) stay to reduce physical deconditioning and to better monitor his neurology. In addition, regular monitoring of possible MCS-related complications, such as haemolysis and thrombosis, was started with daily blood tests [plasma-free haemoglobin, lactose dehydrogenase (LDH), and D-dimer].

During the following days, the inotropic support was decreased; however, infusion of two inotropes remained necessary (milrinone and adrenaline). The clinical condition stabilized, and MCS weaning trial was attempted unsuccessfully on Day 7 of MCS support. It became clear that recovery would not be an option. Therefore, our team contacted his local transplant centre in Poland, who agreed to take over his care and explore transplant candidacy in Poland, as not eligible in the UK.

After extensive MDT discussion, it was decided that the safest way to repatriate the patient would be a fixed-wing transfer on the current ECPELLA support. The transfer was planned following our standard operating procedure for air retrieval of patients on ECMO, which included pre-brief definition of roles, calculation of estimated oxygen use, ensuring supply of double the amount for redundancy, and pre-departure checklist. The patient was electively intubated for the journey and transferred by road to a local airport by a four-person team consisting of consultant intensivist and ITU nurse trained in ECMO retrieval, Impella specialist nurse, and clinical perfusionist.

**Figure 4:**
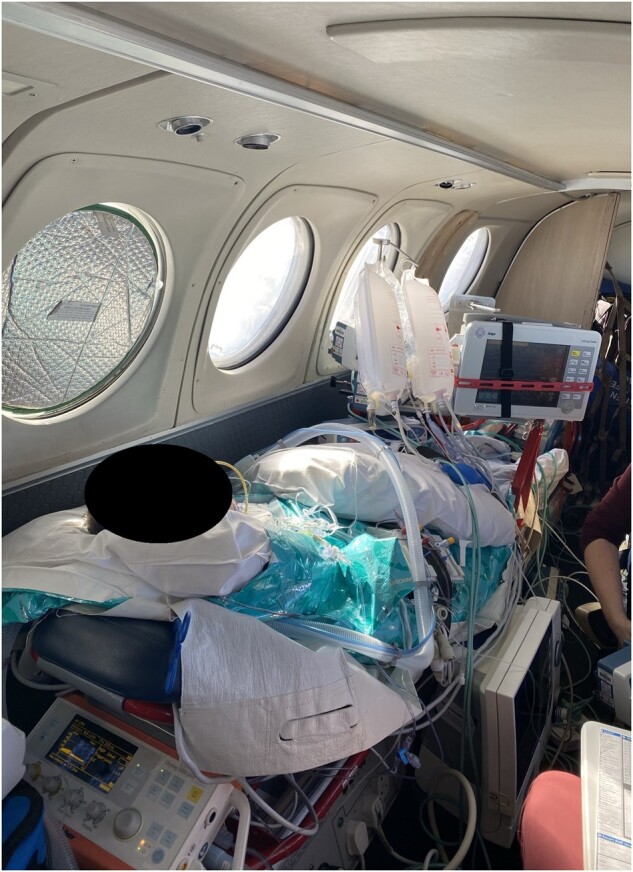
Photograph of patient and equipment layout during the air transfer on a King Air 200 fixed-wing airplane.

At the airfield, the patient was loaded into a King Air 200 fixed-wing airplane and secured into the aircraft with the transport ventilator behind his head, the infusions and monitoring on a bridge over his legs, the Impella console at his side, and the ECMO console by his feet (*Figure 4*). The team had a second Impella console and an ECMO hand crank as well as all usual emergency transfer equipment.

Before take-off, ECMO flows were stable at 3.4 L/min and Impella on P5 providing 2.5 L/min. Sweep gas was matched with the ECMO flows, giving 3 L at 100% O_2_. During take-off, the Impella flows temporarily dropped, and a suction alarm was displayed; however, this rectified without intervention. The ECPELLA flows, oxygenation, and CO_2_ remained stable for the rest of the flight, and medication or MCS titration was not required. The flight lasted about 3 h and covered an 879 mile distance. After landing in Poland, the patient was transferred by road to the receiving hospital where structured handover and post-handover checklist was completed. He was taken directly to the operating theatres (OT) and systematically established on their equipment. Correct position of the devices was confirmed with echocardiogram before leaving the OT.

During the following hospital stay, the patient did not experience any MCS-related complication (bleeding, thrombosis, or haemolysis); he was accepted onto the heart transplant list and escalated to biventricular central MCS (Levitronix CentriMag, Zurich, Switzerland) as more durable short-term support. After 1 month, he was successfully transplanted and discharged home after an uneventful post-operative period. The patient is clinically stable at 12-month follow-up. The endomyocardial biopsies performed within the first year excluded any early rejection episode, and the sequential echocardiograms confirmed preserved and stable graft function.

## Discussion

This case is a great example of the challenges that medical professionals face in treating international patients who require AHF therapies. Even though the initial acute treatment can be tackled with ITU medical optimization and MCS, the longer-term treatment of such patients can be a challenge as their eligibility for AHF therapies crosses nationwide borders.

## Support with multiple mechanical circulatory support devices

Cardiogenic shock remains a major cause of in-hospital mortality,^4^ and MCS devices can be placed emergently to restore organ perfusion.^5^ Veno-arterial extracorporeal membrane oxygenator does not provide a direct unloading effect and can increase LV afterload.^6^ Effective LV unloading prevents pulmonary congestion and contributes to myocardial recovery^7^; therefore, the combination of devices can be effective in improving cardiovascular physiology and possibly survival,^8^ at the expense of higher complication rate.^9^

## Mechanical circulatory support long-distance transfer

Extracorporeal membrane oxygenator retrieval and short-distance transfer to high expertise and tertiary centres is a well-established practice to provide better chances of survival to CS patients.^10,11^

In the last few years, reports on worldwide experience of long-distance air transport have been published.^2,3,12–14^ Guirgis^14^ was the first to introduce the concept of international ‘spoke-to-hub’ network (from Japan to Canada) to increase availability of AHF therapies. Charon’s^2^ group showed the feasibility of long-distance medical evacuation from the remote region of Reunion Island to tertiary centres able to provide heart transplantation in France. Kang^13^ reported his valuable experience with fixed-wing aircraft transport across the USA. In addition to the remarkable long distance covered with no adverse in-transit events, his group reported the first positive experience of two patients transported by ECPELLA. The interesting Yao’s^3^ review showed feasibility and safety of long-distance transport in this cohort of high-risk patients and stressed the important concept of adequate training and planning to avoid complications.

However, publication bias needs to be considered; in fact, unsuccessful cases are most likely to be underreported.

To our knowledge, our case is the first in Europe international air long-distance transport of a patient supported by ECPELLA. Our experience links together the current challenges in the evolving AHF strategies and the increased inter-facility cooperation. Both these clinical and logistic challenges appear to lead to possible improved outcomes, after appropriate assessment, training, and planning. Our experience provides useful information on feasibility of long-distance transport of patients supported by ECPELLA in Europe; however, a wider cohort would be necessary to further comment on positive outcomes.

## Data Availability

No new data were generated or analysed in support of this case report.

## Lead author biography

Francesca Fiorelli is a cardiology consultant specialized in advanced and acute heart failure. She completed her cardiology training and PhD research project in Clinical Physiopathology in Pisa University, Italy. Dr Fiorelli subsequently expanded her expertise in AHF and transplant, working with the transplant teams in Harefield Hospital in London and Queen Elizabeth Hospital in Birmingham, UK. Dr Fiorelli is passionate of cardiovascular haemodynamics, MCS, and cardiac critical care.

**Consent:** The authors confirm that written consent for submission and publication of this case report including images and associated text has been obtained from the patient in line with the Committee on Publication Ethics (COPE) guidance.

**Funding:** None declared.
